# Revealing Within-Agency Variation in Medicare Home Health Quality Ratings

**DOI:** 10.1093/geroni/igaf122.3840

**Published:** 2025-12-31

**Authors:** Shekinah Fashaw-Walters, Olga Jarrín, Anum Zafar

**Affiliations:** University of Pennsylvania, Philadelphia, Pennsylvania, United States; Rutgers, The State University of New Jersey, New Brunswick, New Jersey, United States; Rutgers, The State University of New Jersey, New Brunswick, New Jersey, United States

## Abstract

The Centers for Medicare & Medicaid Services (CMS) publicly reports Home Health Agency (HHA) quality ratings using a composite 5-star system based on seven assessment- and claims-based quality indicators. These ratings are intended to guide consumer decision-making and encourage performance improvement among providers. However, because the ratings reflect agency-level averages, they may mask important variation in the quality of care delivered to different patient subgroups. This study examines within-agency variation in reported performance using national Medicare administrative, assessment, and claims data. We applied CMS’s published star rating methodology to simulate overall and group-specific quality ratings and assess the reliability and informativeness of disaggregated quality ratings. Our findings show that agencies with average to high overall ratings (3–5 stars) can exhibit substantial variation in care outcomes across patient subgroups. This variability is obscured in the reported rating, limiting its utility for some consumers and stakeholders, typically those from marginalized groups. By identifying patterns of intra-agency variation, this analysis highlights opportunities to enhance the information provided by publicly reported quality measures to increase usability for all patient groups. These refinements could better equip consumers to make informed choices and support data-driven improvement efforts among providers. This work demonstrates the feasibility of generating more detailed quality information using existing administrative data sources and methods. These findings provide timely insights into how quality measurement tools can evolve to capture meaningful variation across different patient populations—enabling a clearer understanding of what works best, for whom, and under what conditions.

